# Omalizumab efficacy in cases of chronic spontaneous urticaria is not explained by the inhibition of sera activity in effector cells

**DOI:** 10.1038/s41598-017-09361-4

**Published:** 2017-08-21

**Authors:** Eva Serrano-Candelas, Rubén Martínez-Aranguren, Olga Vega, Gabriel Gastaminza, Joan Bartra, Maria Teresa Audicana, Jorge M. Núñez-Córdoba, Jaime Algorta, Antonio Valero, Margarita Martin, Marta Ferrer

**Affiliations:** 1 Biochemistry Unit, Faculty of Medicine, University of Barcelona, Casanova 143, Barcelona, 08036 Spain; 2 Laboratory of Clinical and Experimental Respiratory Immunoallergy, IDIBAPS, Barcelona, Spain; 3 Department of Allergy and Clinical Immunology, Clinica Universidad de Navarra, Pamplona, Spain; 4 Department of Pneumology and Allergology, Immunoallèrgia Respiratòria Clínical Experimental, IDIBAPS, Hospital Clínic, Barcelona, Spain; 5 Allergy Service, Hospital Santiago, Vitoria, Spain; 6 Research Support Service, Central Clinical Trials Unit, Clinica Universidad de Navarra, Pamplona, Spain; 7 Department of Biochemistry and Molecular Biology, Universidad del Pais Vasco-EHU, Bizkaia, Spain; 8 Centro de Investigación Biomédica en Red de Enfermedades Respiratorias [Biomedical Research Networking Centre on Respiratory Diseases (CIBERES)], Madrid, Spain

## Abstract

Omalizumab (OmAb) is a humanized anti-IgE antibody approved for the treatment of chronic spontaneous urticaria (CSU). OmAb’s mechanism of action is known to include actions on free IgE and on pre-bound IgE. However, OmAb is equally and rapidly effective against autoimmune and non-autoimmune urticaria where IgE involvement is not clear, suggesting the involvement of additional mechanisms of action. In this study, we sought to investigate the ability of OmAb to inhibit mast cell and basophil degranulation induced by sera from CSU patients. For this purpose, we performed a comparison between the *in vitro* incubation of sera from CSU patients treated with OmAb and the *in vivo* administration of OmAb in a clinical trial. We found that OmAb added *in vitro* to sera from CSU patients did not modify the ability of the sera to induce cell degranulation. Similarly, the sera from patients treated with OmAb in the context of the clinical trial who had a good clinical outcome maintained the capacity to activate mast cells and basophils. Thus, we conclude that the beneficial activity of OmAb does not correlate with the ability of patient sera to induce cell degranulation.

## Introduction

Omalizumab (OmAb) is a biological drug that specifically recognizes IgE at the same epitope where IgE is bound to its high-affinity receptor, FcεRI. In addition to its ability to sequester free IgE, it has been demonstrated that OmAb is also capable of accelerating the dissociation of pre-bound IgE in basophils^1, 2^. Our recent data suggest that this also occurs in mast cells and confirm previous basophil data at physiological dose ranges (30–100 µg/ml, 0.2–0.67 µM) in a time- and dose-dependent manner^3^. In these conditions, OmAb was able to inhibit early IgE-triggered events, such as phosphorylation of PLCγ, LAT and Syk, as well as phosphorylation of ERK and later events, such as upregulation of CD63 and leukotriene synthesis^3^. This result explains the effects of OmAb on sustained inflammation in asthmatic patients^4^.

OmAb has recently been approved for chronic spontaneous urticaria (CSU) and has shown high rates of complete control^5^. CSU is a severely disabling disease^6^ defined by the spontaneous onset of wheals, with or without angioedema, persisting for ≥6 weeks. Despite its impact on patient quality of life and morbidity, CSU has an elusive physiopathology^7^. It is widely accepted that CSU has an autoimmune component^8^, wherein dermal mast cells and basophils in CSU patients are triggered by circulating IgE against autoantigens^9^, by IgG against FcεRI^10, 11^ or by IgG against IgE itself^12^, which would be present in the sera of CSU patients. These antibodies may eventually activate mast cells and basophils, causing histamine release^11^ and increased expression of activation markers such as CD63^13^ or CD203c^14^. However, the presence of reactive IgE/IgG has not been observed in approximately half of CSU patients, and, from a clinical standpoint, autoimmune and non-autoimmune CSU cases are indistinguishable from one another. In fact, OmAb is effective in the majority of CSU patients regardless of the presence or absence of autoantibodies. Moreover, in some cases, OmAb is able to cause symptom remission in a very short timeframe, which cannot be explained by the currently postulated mechanisms of action of OmAb^15^.

In an attempt to better understand the mechanisms of action of OmAb in CSU and, more importantly, to better understand the pathophysiology of this disease, we studied the influence of OmAb on the ability of CSU sera to activate mast cells and basophils. Our research was performed in two ways. First, we studied the effects of OmAb addition *in vitro* by pre-incubating sera from CSU patients with OmAb and assessing its ability to modulate basophil and mast cell activation induced by such sera. Second, we determined whether the ability of sera from CSU patients to activate mast cells and primary basophils is altered after OmAb treatment in the context of a clinical trial. We also evaluated whether the levels of histamine, tryptase and C-reactive protein in sera from CSU patients change during treatment to evaluate their use as potential markers for the efficacy of OmAb treatment.

## Results

### Sera from CSU patients differentially induce mast and basophil cell degranulation

**Figure 1:**
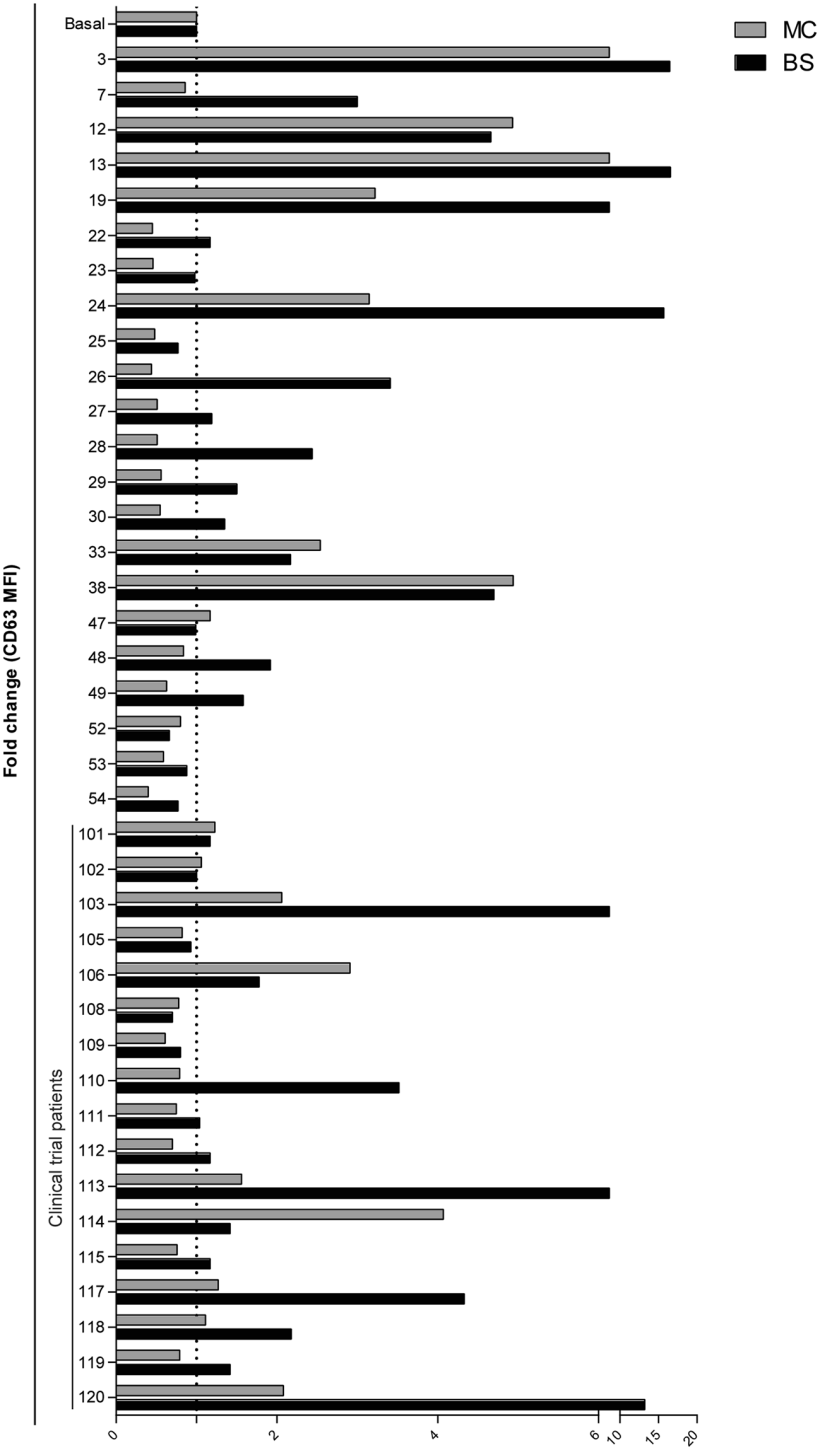
Sera from CSU patients differentially activate mast cells and basophils. (A) Sera from 39 CSU patients were incubated for 30 min with LAD2 cells or *ex vivo* basophils to evaluate their ability to increase CD63 expression in the cell membrane. The bar graph represents fold increases of CD63 (mean fluorescence intensity) on LAD2 cells (MC; grey bars) and *ex vivo* basophils (BS; black bars) compared with that on unstimulated cells. The vertical dashed line corresponds to a fold change value of 1. The mean CD63 on the cell surface of both cell types was correlated (Pearson’s coefficient 0.730; p-value 8.76 × 10^−8^). Patients enrolled in the clinical trial are indicated in the figure.

Thirty-nine CSU patients (22 women and 17 men, mean age: 44 ± 12.2 years) with a median disease duration of 6.7 years were enrolled in the study. Sera from all patients were collected at the beginning of the study. To determine the activating capacity of sera from these CSU patients, we assessed its capacity to induce mast cell and basophil degranulation by analyzing CD63 expression on mast cells (LAD2) and basophils of healthy donors by flow cytometry using the basophil activation test (BAT)^16^. Our results revealed that mast cell and basophil sera-dependent degranulation follows the same pattern (Fig. 1) (Pearson’s coefficient 0.730; p-value 8.76 × 10^−8^). However, the ability of sera from CSU patients to induce cell degranulation is very heterogeneous among different sera. Importantly, this degree of variability in the cell response to sera activity is not attributable to clinical differences between patients.

### Sera from CSU patients incubated with OmAb *in vitro* do not inhibit cell degranulation

**Figure 2:**
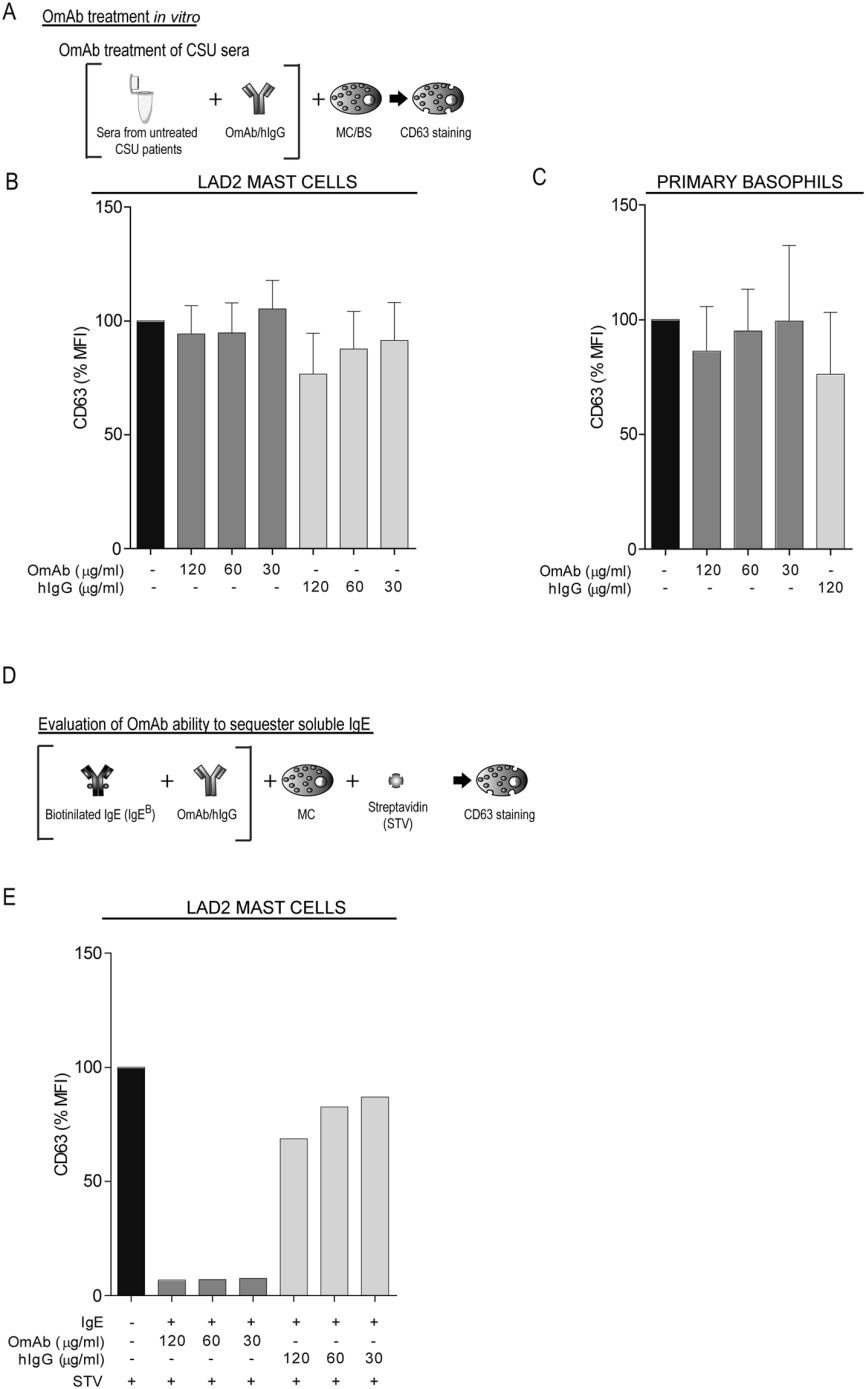
Preincubation of sera from CSU patients with OmAb does not modify the ability of the sera to stimulate mast cells. (**A**) Brief scheme of the experimental protocol used in (**B** and **C**). (**B**) LAD2 cells were incubated with sera samples from CSU patients who were pre-incubated with different doses of OmAb, or hIgG as a control, for 30 min. The bar charts represent the percentage of the mean fluorescence intensity (MFI) of CD63 staining relative to that in OmAb/IgG untreated cells (first bar). (**C**) Basophils from healthy donors were incubated with sera samples from CSU patients who were pre-incubated with different doses of OmAb, or hIgG as a control, for 30 min. The bar charts represent the percentage of the MFI of CD63 staining relative to that in OmAb/IgG untreated cells (first bar). (**D**) Brief scheme of the experimental protocol used in (**E**). (**E**) IgE^B^ was incubated with different doses of OmAb, or IgG as a control, for 30 min. Then, LAD2 cells were incubated overnight with this mixture, and cells were stained for CD63 after IgE^B^ challenge. The bar charts represent the percentage of the MFI of CD63 staining relative to that in OmAb/IgG untreated cells (first bar).

To test the ability of OmAb to sequester soluble factors present in CSU or to hinder their activating capacity, we selected several CSU sera able to induce cell degranulation of LAD2 cells and basophils and treated them with different concentrations of OmAb or human IgG (hIgG) as a control (Fig. 2A). These pre-treated sera were subsequently incubated with LAD2 mast cells or primary basophils, and CD63 surface expression was evaluated. Our results showed that OmAb does not inhibit the ability of these sera to induce degranulation of LAD2 cells (Fig. 2B) or primary basophils (Fig. 2C). To test whether OmAb was fully active (i.e., able to sequester soluble IgE), the same OmAb concentrations and procedures were used with IgE^B^ and subsequent triggering with streptavidin (STV), achieving a near total inhibition of mast cell degranulation (Fig. 2D,E).

Altogether, our data indicate that OmAb effectively prevents IgE from binding to FcεRI, preventing IgE-mediated cell degranulation, but is unable to inhibit CSU patient sera-dependent degranulation in mast cells and basophils under the same conditions.

### Clinical outcomes from the OmAb clinical trial

**Figure 3:**
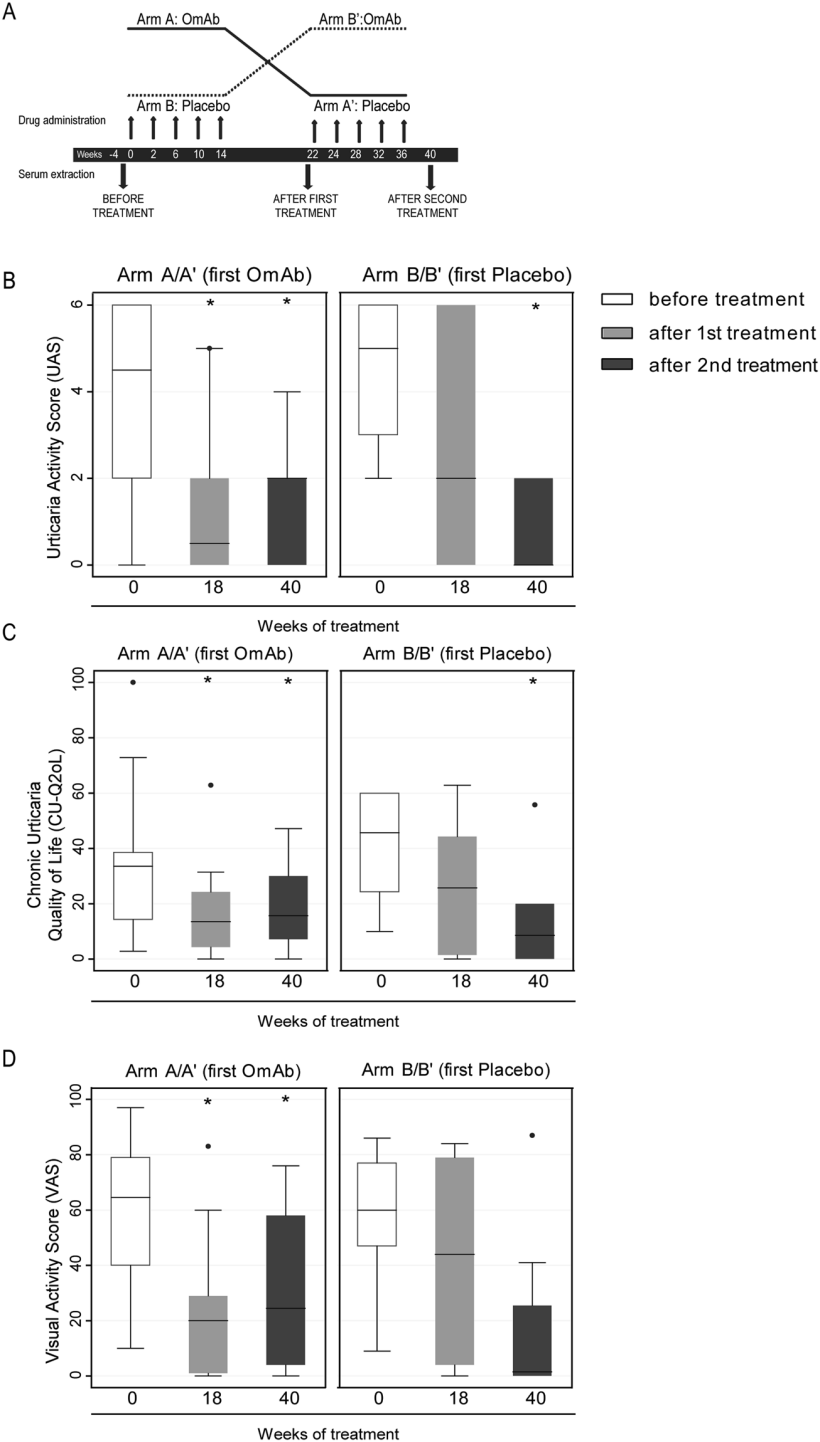
Clinical trial study. (**A**) Scheme of the double-blind, placebo-controlled, randomized, cross-over (2 × 2) clinical trial. (**B**) Urticaria activity score (UAS), (**C**) Chronic Urticaria Quality of Life (CU-Q2oL) score, and (**D**) Visual Activity Scale (VAS) score reported by patients before the start of the study and after the two treatment periods (weeks 0, 18 and 40, respectively). The first three bars represent the patients who received OmAb followed by placebo (Arm A/A’), and the second three bars represent the patients who received placebo followed by OmAb (Arm B/B’). The box plots represent the median, 25th percentile and 75th percentile of UAS/CU-Q2oL/VAS scores of 17 patients enrolled in the clinical trial before and after receiving OmAb or placebo. The outer point corresponds to a patient who did not respond to the OmAb treatment. The Wilcoxon matched-pairs signed-rank test was used to evaluate differences between the values at baseline and at the end of the study (*p < 0.05).

Of the 39 CSU patients enrolled in the study (Supplementary Table 1), seventeen patients were recruited to participate in a clinical trial to analyze the efficacy of OmAb in the treatment of CSU. Fig. 3A shows a brief scheme of the clinical trial design, which was structured as a cross-over study. Of these 17 patients (retention rate: 85%), 10 started in the OmAb arm (Arm A) and 7 started in the placebo arm (Arm B). The mean patient age was 46.7 (SD: 12.5) years, and 9 (52.9%) were women.

The Urticaria Activity Score (UAS) changes from baseline to the end of the study revealed a greater average improvement in response to OmAb treatment (mean UAS reduction = −2.4, SD = 2.5; median UAS reduction = −2; p25 = −4, p75 = 0) than in response to placebo (mean UAS reduction = −1.2, SD = 2.6; median UAS reduction = −2; p25 = −3, p75 = 1); however, the difference between the placebo and OmAb treatment groups was not statistically significant (p = 0.380). In contrast, OmAb treatment resulted in a statistically significant improvement in the post-treatment UAS compared with the baseline UAS (baseline UAS: 3; p25 = 2, p75 = 6; post-OmAb UAS: 0; p25 = 0, p75 = 2; p = 0.004) (Fig. 3B; Supplementary Table 2A,B).

Comparisons of the change in the chronic urticaria quality of life (CU-Q_2_OL) score from baseline to the end of the study showed a greater average improvement with OmAb treatment (mean overall score reduction = −13.0, SD = 22.0; median overall score reduction = −5.4; p25 = −29.3, p75 = 1.1) than with placebo (mean score reduction = −7.0, SD = 15.0; median overall score reduction = −4.9; p25 = −16.8, p75 = 3.3), although the difference was not statistically significant (p = 0.339). OmAb treatment resulted in a statistically significant improvement in the post-treatment score compared with the baseline score (baseline overall score: 22.8; p25 = 8.7, p75 = 34.8; post-OmAb treatment overall score: 6.5; p25 = 3.3, p75 = 15.2; p = 0.047) (Fig. 3C; Supplementary Table 2A,B).

The comparison between the change in the visual analogue scale (VAS) score from baseline to the end of the study showed a greater average improvement with OmAb treatment (mean VAS reduction = −29.8, SD = 29.4; median VAS reduction = −23; p25 = −53, p75 = −7) than with placebo (mean VAS reduction = −9.5, SD = 41.7; median VAS reduction = −11; p25 = −45, p75 = 21), but the difference was not statistically significant (p = 0.218). OmAb resulted in a significantly improved VAS score post-treatment compared with baseline (baseline VAS: 60; p25 = 25, p75 = 75; post-OmAb VAS: 10; p25 = 1, p75 = 29; p = 0.001) (Fig. 3D; Supplementary Table 2A,B).

### Sera from CSU patients from the clinical trial maintained its ability to activate mast cells and basophils

The results described above indicate that although OmAb treatment showed high efficacy in the control of CSU, incubation of sera from CSU patients with OmAb *in vitro* did not hinder the ability of the sera to activate mast cells (LAD2) and basophils. For this reason, we aimed to determine whether the *in vivo* administration of OmAb had any impact on the activating capacity of patient sera. To test this hypothesis, we used sera from CSU patients collected at different time points during the clinical trial: before treatment (untreated CSU sera), after the first treatment (corresponding to week 18) and after the second treatment (corresponding to week 40).

**Figure 4:**
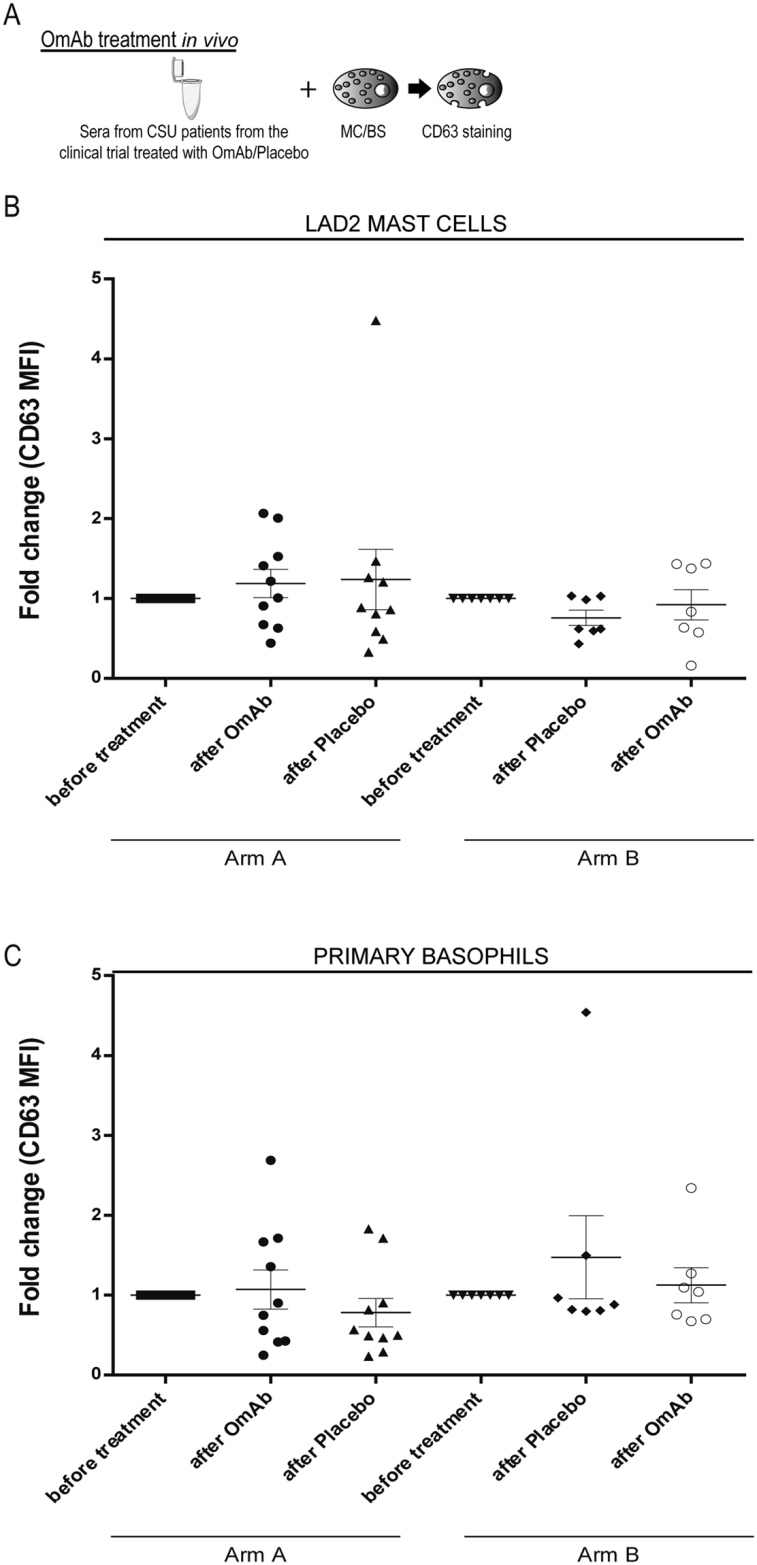
Sera from CSU patients enrolled in the clinical trial, treated with either OmAb or placebo, did not modify the capacity of the sera to activate mast cells or basophils. (**A**) Protocol followed in the assay. (**B**) Dot plot showing fold change of CD63 mean fluorescence intensity (MFI) of LAD2 mast cells and (**C**) basophils incubated with sera from CSU patients at different time points of the clinical trial.

We incubated these CSU sera samples with LAD2 mast cells and basophils and evaluated their activating capacity by measuring CD63 by flow cytometry (Fig. 4A). Our results show that their sera maintained the same activating capacity for mast cells (Fig. 4B) and basophils (Fig. 4C) throughout the study. It is important to note that all patients, regardless of the ability of their serum to activate basophils and mast cells, had similar positive clinical outcomes with OmAb treatment.

These results show that the capacity of CSU sera to activate mast cells and basophils is not a good marker for predicting the clinical response to OmAb treatment.

### The histamine, tryptase and C-reactive protein levels present in the sera of CSU patients are maintained following OmAb treatment

**Figure 5:**
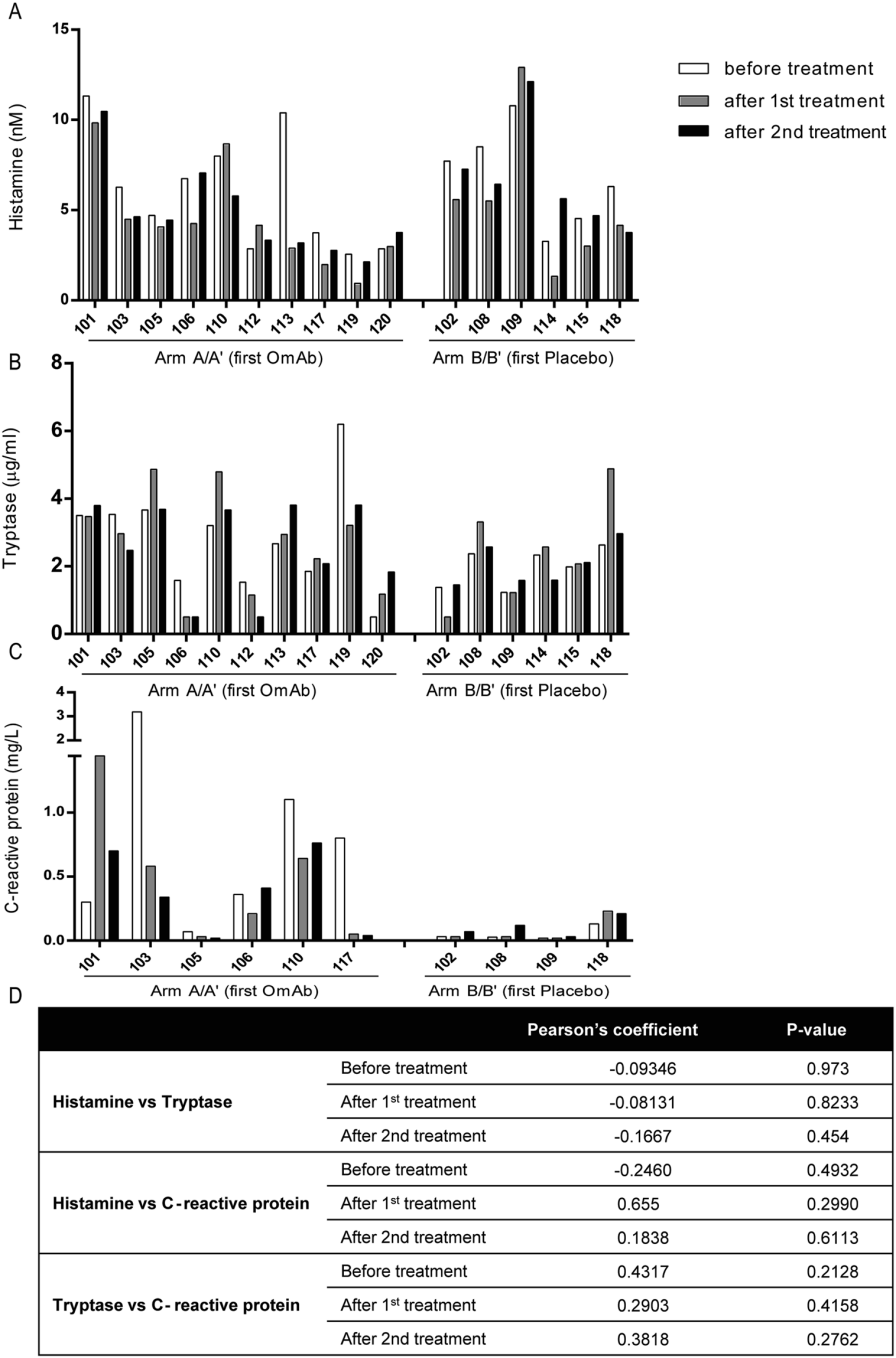
Histamine, tryptase or C-reactive protein levels in sera from CSU patients did not change after OmAb treatment. The charts represent the concentration of histamine (**A**), tryptase (**B**) and C-reactive protein (**C**) in sera from CSU patients at different time points of the OmAb/placebo treatment. (**D**) Table showing Pearson’s coefficients for the different substances tested.

Because the analysis of the activating capacity of sera from CSU patients for mast cells and basophils was not useful for predicting the response to OmAb treatment, we sought to determine whether other potential mediators or proinflammatory markers present in the sera changed during treatment, as they may serve as markers of symptom remission. We evaluated the levels of histamine, tryptase and C-reactive protein in sera from CSU patients during treatment. As shown in Fig. 5, sera from CSU patients initially contained variable amounts of these substances, which had no correlation with clinical outcome, and the amounts of these soluble factors did not vary throughout the treatment course. Moreover, the concentrations of these markers did not seem to be correlated with each other (Fig. 5D).

Other standard practices reported in the literature include the evaluation of the β-hexosaminidase activity of cell supernatants after incubation of CSU sera with mast cells^17, 18^. Given the results found for histamine, we sought to evaluate the β-hexosaminidase content in sera from CSU patients compared with that in sera from healthy controls. We found that both sera samples had considerable intrinsic enzymatic activity (Supplementary Fig. S1A). Moreover, the evaluation of β-hexosaminidase content in CSU samples during treatment showed no change with either OmAb or placebo treatment (Supplementary Fig. S1B). Thus, we concluded that this colorimetric approach to measuring cell degranulation is not valid to assess sera-dependent mast cell degranulation. Moreover, the variability in sera colors, despite subtraction of the background, can also alter the assay results.

## Discussion

OmAb has been postulated to be effective in the treatment of CSU due to its ability to sequester free IgE and, indirectly, to downregulate FcεRI^19–21^. Benefits of omalizumab treatment (300 mg) are commonly evident early (before week 4) in CSU patients and persisted to week 24^5, 22, 23^. However, in some cases its clinical effectiveness is evident much earlier than downregulation of the IgE receptor could take place, given that there are patients whose symptoms resolve as early as 24 or 48 h after OmAb administration^24, 25^. In certain cases, the effectiveness of OmAb is thought to be associated with the blocking of autoreactive IgE antibodies^9^, although the presence of these autoantibodies cannot be found in all CSU patients. In a recent review is summarized that OmAb therapy is successful in patients with or without a positive test for IgG autoantibodies against FcεRI or IgE, or IgE autoantibodies against thyroperoxidase^15^. In this study, we aimed to further explore the action of OmAb in CSU patients and to better understand the pathophysiology of this disease.

We first explored the capacity of sera from CSU patients to activate mast cells (LAD2) and primary basophils. It has been reported that between 5 and 10% of CSU patients present with specific autoantibodies against IgE itself^12, 26^. Using LAD2 cells as a mast cell model permits the uniform evaluation of serum activation in a context where FcεRI is not occupied. Therefore, this model allows us to discard anti-IgE antibodies as the soluble factor present in the sera responsible for cell degranulation, as LAD2 cells are not IgE-sensitized.

To explore the action of OmAb in CSU patients, we analyzed the ability of OmAb to block the serum-activating ability of sera from CSU patients to induce mast cell (LAD2) and basophil degranulation. We used ranges of OmAb doses close to physiological concentrations *in vitro* for comparison with the data obtained from the sera from CSU patients treated with OmAb in a clinical trial.

From our *in vitro* experiments, we can conclude that OmAb did not interfere with or block the serum factor or antibody responsible for activating mast cells and basophils. This result highlights the fact that IgE is not the activating factor present in the sera from our patients. Consistent with this result, the use of quilizumab, a monoclonal antibody that binds to the M1-prime segment of the IgE membrane causing IgE-switch depletion and cell apoptosis, did not prove to be effective in a clinical trial involving 45 patients with CSU^27^.

We next analyzed the efficacy of OmAb in the treatment of CSU in a clinical trial. OmAb treatment was efficacious in all CSU subtypes, regardless of their autoimmune and non-autoimmune behavior, defined as the ability of the sera to activate basophils and mast cells. Consistent with these findings, it has been recently published that serum reactivity can be useful for predicting the time to response to OmAb^28^. The authors concluded that patients with a positive autologous skin serum test (ASST) respond faster to OmAb, but they determined that the final OmAb efficacy did not depend on the result of the ASST, which is in accordance with our results demonstrating no differences in OmAb effectiveness regardless of the activating capacity of sera from the patients enrolled in the clinical trial. This result is in accordance with the clinical experience of others^29, 30^. Our study shows a statistical significant difference between the baseline to OmAb post-treatment. However there is not significant difference between placebo and OmAb treatment although we observed a clear improvement. While not statistically significant, the magnitude of this improvement may have clinical relevance and should not lead these results to be discarded. The residual effect of OmAb in the Arm A/A’ may have contributed to alter significance. In addition, the impact of the placebo effect should not be discounted. This symptom reduction in the placebo group has also been observed in other OmAb efficacy studies that evaluated the mean change from baseline in symptom scores^5^.

Our results also reveal that the activating capacity of sera from CSU patients did not change after treatment with OmAb. This result is in accordance with that of other studies that did not reveal changes in ASST in CSU patients during remission^31, 32^. In the proof of concept study for the use of OmAb in CSU, Kaplan^19^ treated 12 CSU patients and analyzed the ability of sera to activate basophils before and after administering OmAb. In patients with complete resolution of or remitted symptoms, they observed a trend toward the induction of a lower histamine release than that resulting from the same sera at the beginning of the treatment. However, they did not find a clear correlation between symptom remission and serum activity, with patients who exhibited complete responses maintaining the same serum activity and vice versa. Moreover, as we will discuss below, the analysis of histamine release can yield artificial results due to the serum content of histamine in these patients.

Finally, we also describe the intrinsic levels of the soluble mediators ß-hexosaminidase, histamine, tryptase and C-reactive protein in the sera from CSU patients. The concentrations of these soluble factors were not observed to be associated with either the clinical outcomes or the responses to treatment. We conclude that the use of β-hexosaminidase analysis to determine the ability of patient sera to induce mast cell degranulation could result in artificial values that are determined more by sera activity than by cell activity itself. These results also call into question the use of the ASST in the evaluation of autoimmune CSU patients, as soluble factors such bradykinin, complement fragments or cytokines may trigger skin mast cell activation and yield false-positive results^33^.

We should also consider that removal of IgE from the mast cell and basophil surface may desensitize them and would leave them unable to be activated by IgE or anti-IgE autoreactive antibodies. However, IgE or anti-IgE antibodies are not the unique cause of CSU symptoms and that OmAb is acting through other mechanisms. As highlighted by a recent comprehensive review^15^, there could be alternative mechanisms. Accordingly, it has been demonstrated that OmAb is also effective in several types of inducible urticaria, as solar urticaria^34, 35^, cold- and heat-induced urticaria^36–38^ and delayed pressure urticaria or symptomatic dermographism^39, 40^ where non-IgE-mechanisms have been demonstrated, indicating that OmAb may act beyond IgE-mediated mechanisms. We have recently published a review in which we discuss the action of OmAb cells other than mast cells and basophils^41^. For example, OmAb can target membrane-IgE (mIgE) in IgE^+^ B-cells, reducing IL4R expression and IgE synthesis and decreasing the number of these cells, possibly by causing B-cell anergy^42^. OmAb has also been reported to cause eosinophil apoptosis^43^, a finding that is in agreement with the decrease in eosinophilia found in asthma patients after OmAb administration^44^. Finally, OmAb has been observed to inhibit corneal angiogenesis in IgE-deficient mice, but it failed to inhibit angiogenesis in Fcgr1−/− mice, suggesting a role of OmAb as an IgG^45^.

In conclusion, our data show that OmAb efficacy in cases of CSU is not explained by the inhibition of sera activity in mast cells and basophils and indirectly proves that autoimmunity, understood as the ability of CSU sera to activate basophils and mast cells, is not a good biomarker for predicting clinical CSU responses to OmAb treatment. We also conclude that proinflammatory components present in sera from CSU patients are not useful for predicting OmAb treatment responses. Moreover, these results also suggest a possible direct effect of OmAb, not only in mast cells and basophils but also in other cells, which could explain its effectiveness in CSU and other pathologies.

## Methods

This study was approved by the institutional review board of the University of Navarra. The clinical trial was approved by the Spanish Agency of Medicines and Medicinal Products (AEMPS).

### Cells and reagents

The LAD2 human mast cell line was provided by Drs A. Kirshenbaum and D.D. Metcalfe (National Institutes of Health, Bethesda, MD) and grown in StemPro-34 serum-free medium (Invitrogen Life Technologies, Carlsbad, CA, USA) supplemented with StemPro-34 Nutrient, L-glutamine (2 mM), penicillin (100 U/ml), streptomycin (100 μg/ml), and 100 ng/ml recombinant stem cell factor (SCF) (Amgen, Thousand Oaks, CA, USA). The experiments with basophils were performed as previously reported^46^ using blood from non-atopic donors. Biotinylated human IgE (IgE^B^) was obtained from Abbiotec (San Diego, CA, USA). Streptavidin (STV) was purchased from Sigma (Sigma, St. Louis, MO, USA). Whole-molecule human hIgG was obtained from Jackson ImmunoResearch (West Grove, PA, USA). PerCP-Cy5.5-conjugated anti-human HLA-DR and PE-Cy7-conjugated anti-human CD123 antibodies were purchased from eBioscience (San Diego, CA, USA), and APC-conjugated anti-human CD203c and PE-conjugated anti-human CD63 antibodies were purchased from BD Bioscience (Mountain View, CA).

### CSU patients

Thirty-nine patients were enrolled in this study. All patients had been diagnosed with a 2-month to 40-year history of CSU, and 31 (79%) suffered from angioedema along with urticaria. Supplementary Table 1 in the online supporting information includes demographic and clinical data of these patients. All patients provided their written consent to participate in this study, and 17 (43.5%) were included in the clinical trial described below.

### Ethics statement

The study design and protocol was approved by the Food and Drug Spanish Agency (AEMPS), the State Ethics Committee and the Ethics Research Committee of the University of Navarra. The authors performed these procedures in accordance with the approved guidelines, obtaining informed consent from each subject before conducting the experiments.

### Mast cell and basophil activation with sera from CSU patients

LAD2 cells (0.2 × 10^6^) were re-suspended in Tyrode’s buffer^47^ and incubated for 30 minutes at 37 °C with 25 μl of sera from CSU patients in a final volume of 60 μl. Cells were collected for CD63 testing using a FACSCalibur flow cytometer (FACScan; BD Biosciences). A total of 100 µl of blood from healthy donors was pre-incubated with 20 µl of stimulation buffer containing IL-3 at 37 °C for 10 minutes. Thereafter, blood was stimulated with 80 µl of sera. After 30 min at 37 °C, stimulation was stopped and the serum was eliminated by washing the samples with cold washing buffer (PBS, 2 mM EDTA). Samples were stained with HLA-DR, PerCP-Cy5.5, CD123 PE-Cy7, CD203c APC and CD63 PE for 30 min at 4 °C. Erythrocytes were lysed for 15 min at RT, and samples were washed twice prior to separating them in a FASCCanto II flow cytometer (FACScan). Data were analyzed with FlowJo Tree Star software (Ashland, OR, USA). In all cases, dead cells were eliminated based on their forward (FSC) and side scattering (SSC) profiles.

### *In vitro* preincubation of sera from CSU patients or IgE with OmAb/IgG and LAD2 and primary basophil stimulation

Sera from CSU patients were treated for 30 min at 37 °C with OmAb or hIgG, as an immunoglobulin control, at different concentrations in Tyrode’s buffer. Then, LAD2 cells and basophils from donors were stimulated with these pre-treated sera as described above, and CD63 was assessed by flow cytometry. For IgE-dependent LAD2 degranulation, biotinylated IgE (IgE^B^) was incubated with different concentrations of OmAb/hIgG for 30 min at 37 °C. Then, LAD2 mast cells were incubated overnight with this mixture at 37 °C, and, after extensive washing, cells were stimulated with streptavidin for 30 min. CD63 was assessed by flow cytometry.

### Clinical trial

The multicenter, double-blinded, placebo-controlled, randomized, cross-over clinical trial of OmAb was registered at Clinical Trials.gov (NCT01713725). Twenty participants were recruited from those included in the study. These patients were randomly assigned to receive OmAb or a matching placebo for a total of 14 weeks (first period). A total of 300 mg of OmAb was subcutaneously administered every two weeks during the first four weeks and every 4 weeks thereafter. After a washout period of 4 weeks, patients were crossed over to the other treatment for an additional 14 weeks (second period). Patients were followed-up for 12 weeks after the end of the second period.

### Disease activity and quality of life assessment

The urticaria activity score (UAS) was used to evaluate the main CSU characteristics (pruritus and number of wheals), with a total daily score ranging from 0 to 6 (lowest to highest disease activity)^48^. The validated Spanish version of the Chronic Urticaria Quality of Life Questionnaire (CU-Q_2_OL) was used to measure 6 CSU-specific dimensions of health-related quality of life: pruritus (2 items), impact on life activities (6 items), sleep disorders (5 items), limitations (3 items), appearance (5 items), and swelling (2 items)^49^. Scores were transformed to a 0 to 100 scale, with higher scores representing a worse quality of life. Additionally, the visual analogue scale (VAS) was used as a complementary measure of CSU severity. The VAS score was determined by measuring the distance in millimeters (mm) between the left corner of a straight, 100-mm horizontal line and the mark indicated on the line by each patient.

### LAD2 and primary basophil stimulation with sera from CSU patients treated with OmAb/Placebo

Patient sera samples from the clinical trial phases were obtained at the start of the study, after administering 5 OmAb or placebo doses (week 18) and at the end of the study (week 40). These sera samples were used to assess cell degranulation of mast cells and basophils as detailed above.

### Measurement of soluble substances in sera from CSU patients

Histamine, tryptase and C-reactive protein levels in sera were measured using the EIA Histamine kit (Immunotech, BeckmanCoulter, Marseille, France), the enzyme immunoassay ImmunoCAP FEIA (Thermo Fisher, Uppsala, Sweden) and the C-reactive protein High Sensitivity immunoturbidimetric assay (Roche Diagnostics, Mannheim, Germany), respectively, following the manufacturers’ instructions. Measurement of intrinsic β-hexosaminidase from CSU sera was performed by incubating sera with substrate (p-nitrophenyl-N-acetyl-β-D-glucopyranoside) for 30 min and, after stopping the reaction, measuring the absorbance at 405 nm.

### Statistical data analysis

The results of the *in vitro* assays are expressed as the means ± standard deviations of the mean value (SD). One-way ANOVA or unpaired t-tests were used to determine significant differences (p-value), and Pearson’s coefficient was used to analyze the correlation between different parameters using GraphPad software. The Wilcoxon matched-pairs signed-rank test was used to compare efficacy parameters according to interventions using Stata 14 (StataCorp LP, College Station, TX). Asterisks indicate the following p values: *p ≤ 0.05, **p ≤ 0.01, ***p ≤ 0.001, and ****p ≤ 0.0001.

## Electronic supplementary material

Supplementary Information

## References

[1] Eggel A (2011). Inhibition of ongoing allergic reactions using a novel anti-IgE DARPin-Fc fusion protein. Allergy.

[2] Pennington LF (2016). Structural basis of omalizumab therapy and omalizumab-mediated IgE exchange. Nat Commun.

[3] Serrano-Candelas E (2016). Comparable actions of omalizumab on mast cells and basophils. Clin Exp Allergy.

[4] Pasha, M. A. *et al*. The effect of omalizumab on small airway inflammation as measured by exhaled nitric oxide in moderate-to-severe asthmatic patients. *Allergy Asthma Proc ***35**, 241–249, doi:10.2500/aap.2014.35.3741 (2014). 10.2500/aap.2014.35.3741 24801467

[5] Maurer M (2013). Omalizumab for the treatment of chronic idiopathic or spontaneous urticaria. N Engl J Med.

[6] Zuberbier T (2014). The EAACI/GA(2) LEN/EDF/WAO Guideline for the definition, classification, diagnosis, and management of urticaria: the 2013 revision and update. Allergy.

[7] Ferrer M (2015). Immunological events in chronic spontaneous urticaria. Clin Transl Allergy.

[8] Konstantinou GN (2013). EAACI taskforce position paper: evidence for autoimmune urticaria and proposal for defining diagnostic criteria. Allergy.

[9] Altrichter S (2011). IgE mediated autoallergy against thyroid peroxidase–a novel pathomechanism of chronic spontaneous urticaria?. PLoS One.

[10] Hide M (1993). Autoantibodies against the high-affinity IgE receptor as a cause of histamine release in chronic urticaria. N Engl J Med.

[11] Ferrer M, Kinet JP, Kaplan AP (1998). Comparative studies of functional and binding assays for IgG anti-Fc(epsilon)RIalpha (alpha-subunit) in chronic urticaria. J Allergy Clin Immunol.

[12] Gruber BL, Baeza ML, Marchese MJ, Agnello V, Kaplan AP (1988). Prevalence and functional role of anti-IgE autoantibodies in urticarial syndromes. J Invest Dermatol.

[13] Wedi B, Novacovic V, Koerner M, Kapp A (2000). Chronic urticaria serum induces histamine release, leukotriene production, and basophil CD63 surface expression–inhibitory effects ofanti-inflammatory drugs. J Allergy Clin Immunol.

[14] Yasnowsky KM (2006). Chronic urticaria sera increase basophil CD203c expression. J Allergy Clin Immunol.

[15] Kaplan AP, Gimenez-Arnau AM, Saini SS (2017). Mechanisms of action that contribute to efficacy of omalizumab in chronic spontaneous urticaria. Allergy.

[16] Hoffmann HJ (2015). The clinical utility of basophil activation testing in diagnosis and monitoring of allergic disease. Allergy.

[17] Tong LJ, Balakrishnan G, Kochan JP, Kinet JP, Kaplan AP (1997). Assessment of autoimmunity in patients with chronic urticaria. J Allergy Clin Immunol.

[18] Bossi F (2011). Mast cells are critically involved in serum-mediated vascular leakage in chronic urticaria beyond high-affinity IgE receptor stimulation. Allergy.

[19] Kaplan AP, Joseph K, Maykut RJ, Geba GP, Zeldin RK (2008). Treatment of chronic autoimmune urticaria with omalizumab. J Allergy Clin Immunol.

[20] MacGlashan D, Xia HZ, Schwartz LB, Gong J (2001). IgE-regulated loss, not IgE-regulated synthesis, controls expression of FcepsilonRI in human basophils. Journal of leukocyte biology.

[21] Beck LA, Marcotte GV, MacGlashan D, Togias A, Saini S (2004). Omalizumab-induced reductions in mast cell Fce psilon RI expression and function. J Allergy Clin Immunol.

[22] Kaplan A (2016). Timing and duration of omalizumab response in patients with chronic idiopathic/spontaneous urticaria. J Allergy Clin Immunol.

[23] Chang TW (2015). The potential pharmacologic mechanisms of omalizumab in patients with chronic spontaneous urticaria. J Allergy Clin Immunol.

[24] Metz, M., Ohanyan, T., Church, M. K. & Maurer, M. Omalizumab is an effective and rapidly acting therapy in difficult-to-treat chronic urticaria: a retrospective clinical analysis. *J Dermatol Sci ***73**, 57–62, doi:10.1016/j.jdermsci.2013.08.011 (2014). 10.1016/j.jdermsci.2013.08.011 24060603

[25] MacGlashan DW (1997). Down-regulation of Fc(epsilon)RI expression on human basophils during *in vivo* treatment of atopic patients with anti-IgE antibody. J Immunol.

[26] Goh, C. L. & Tan, K. T. Chronic autoimmune urticaria: where we stand? *Indian J Dermatol ***54**, 269–274, doi:10.4103/0019-5154.55640 (2009). 10.4103/0019-5154.55640 PMC2810697 20161862

[27] Harris JM (2016). A randomized trial of quilizumab in adults with refractory chronic spontaneous urticaria. J Allergy Clin Immunol.

[28] Gericke J (2017). Serum autoreactivity predicts time to response to omalizumab therapy in chronic spontaneous urticaria. J Allergy Clin Immunol.

[29] Ferrer M (2011). Omalizumab is effective in nonautoimmune urticaria. J Allergy Clin Immunol.

[30] Viswanathan, R. K., Moss, M. H. & Mathur, S. K. Retrospective analysis of the efficacy of omalizumab in chronic refractory urticaria. *Allergy Asthma Proc* **34**, 446–452, doi:10.2500/aap.2013.34.3694 (2013). 10.2500/aap.2013.34.3694 PMC3753597 23998242

[31] Grattan CE, Wallington TB, Warin RP, Kennedy CT, Bradfield JW (1986). A serological mediator in chronic idiopathic urticaria–a clinical, immunological and histological evaluation. Br J Dermatol.

[32] Fusari A, Colangelo C, Bonifazi F, Antonicelli L (2005). The autologous serum skin test in the follow-up of patients with chronic urticaria. Allergy.

[33] Bernstein JA (2014). The diagnosis and management of acute and chronic urticaria: 2014 update. J Allergy Clin Immunol.

[34] Waibel KH, Reese DA, Hamilton RG, Devillez RL (2010). Partial improvement of solar urticaria after omalizumab. J Allergy Clin Immunol.

[35] Guzelbey O (2008). Successful treatment of solar urticaria with anti-immunoglobulin E therapy. Allergy.

[36] Boyce JA (2006). Successful treatment of cold-induced urticaria/anaphylaxis with anti-IgE. J Allergy Clin Immunol.

[37] Bullerkotte U, Wieczorek D, Kapp A, Wedi B (2010). Effective treatment of refractory severe heat urticaria with omalizumab. Allergy.

[38] Metz, M. *et al*. Omalizumab is effective in cold urticaria-results of a randomized placebo-controlled trial. *J Allergy Clin Immunol*, doi:10.1016/j.jaci.2017.01.043 (2017). 10.1016/j.jaci.2017.01.043 28389393

[39] Bindslev-Jensen C, Skov PS (2010). Efficacy of omalizumab in delayed pressure urticaria: a case report. Allergy.

[40] Maurer, M. *et al*. Omalizumab is effective in symptomatic dermographism-results of a randomized placebo-controlled trial. *J Allergy Clin Immunol*, doi:10.1016/j.jaci.2017.01.042 (2017). 10.1016/j.jaci.2017.01.042 28389391

[41] Navines-Ferrer A, Serrano-Candelas E, Molina-Molina GJ, Martin M (2016). IgE-Related Chronic Diseases and Anti-IgE-Based Treatments. J Immunol Res.

[42] Chan MA, Gigliotti NM, Dotson AL, Rosenwasser LJ (2013). Omalizumab may decrease IgE synthesis by targeting membrane IgE+ human B cells. Clin Transl Allergy.

[43] Noga O (2006). Effect of omalizumab treatment on peripheral eosinophil and T-lymphocyte function in patients with allergic asthma. J Allergy Clin Immunol.

[44] Skiepko R (2014). Changes in blood eosinophilia during omalizumab therapy as a predictor of asthma exacerbation. Postepy Dermatol Alergol.

[45] Bogdanovich, S. *et al*. Human IgG1 antibodies suppress angiogenesis in a target-independent manner. *Signal Transduct Target Ther* **1**, doi:10.1038/sigtrans.2015.1 (2016). 10.1038/sigtrans.2015.1 PMC4763941 26918197

[46] Sanz ML (2002). Flow cytometric basophil activation test by detection of CD63 expression in patients with immediate-type reactions to betalactam antibiotics. Clin Exp Allergy.

[47] Alvarez-Errico D (2011). CD84 negatively regulates IgE high-affinity receptor signaling in human mast cells. J Immunol.

[48] Jauregui I (2014). Assessment of severity and quality of life in chronic urticaria. J Investig Allergol Clin Immunol.

[49] Valero A (2008). Adaptation and Validation of the Spanish Version of the Chronic Urticaria Quality of Life Questionnaire (CU-Q(2)oL). J Investig Allergol Clin Immunol.

