# Supplementary material for: Omalizumab efficacy in cases of chronic spontaneous urticaria is not explained by the inhibition of sera activity in effector cells
Source: Sci Rep. 2017 Aug 21;7:8985. doi: 10.1038/s41598-017-09361-4 (PMC5566209; doi:10.1038/s41598-017-09361-4)
Supplement: Supplementary file 1 — Supplementary Information [file 41598_2017_9361_MOESM1_ESM.pdf]

Omalizumab efficacy in cases of chronic spontaneous urticaria is not explained by the inhibition of sera activity in effector cells

Eva Serrano-Candelas, PhD[1,2], Rubén Martínez-Aranguren, PhD[3], Olga Vega, MD, PhD[3], Gabriel Gastaminza, MD, PhD[3], Joan Bartra, MD, PhD[4], Maria Teresa Audicana, MD, PhD,[5] Jorge M. Núñez-Córdoba, MD, PhD[6], Jaime Algorta[7], Antonio Valero, MD, PhD[4,8], Margarita Martin, PhD[1,2]*, Marta Ferrer, MD, PhD[3*]

Affiliations:

[1]Biochemistry Unit, Faculty of Medicine, University of Barcelona, Casanova 143, Barcelona, 08036, Spain. [2]Laboratory of Clinical and Experimental Respiratory Immunoallergy, IDIBAPS, Barcelona, Spain. [3]Department of Allergy and Clinical Immunology, Clinica Universidad de Navarra, Pamplona, Spain. [4]Department of Pneumology and Allergology. Immunoallèrgia Respiratòria Clínica I Experimental, IDIBAPS. Hospital Clínic. Barcelona. [5]Allergy Service, Hospital Santiago, Vitoria, Spain. [6]Research Support Service, Central Clinical Trials Unit, Clinica Universidad de Navarra, Pamplona, Spain. [7]Department of Biochemistry and Molecular Biology, Universidad del Pais Vasco-EHU [8]Centro de Investigación Biomédica en Red de Enfermedades Respiratorias [Biomedical Research Networking Centre on Respiratory Diseases (CIBERES)]

* These authors contributed equally to this paper.

Supplementary Table 1: Characteristics of CSU patients

Patient	Sex	Age	CSU Diagnostic (years)	Angioedema	IgE (UI/L)	W (Kg)	H (cm)	Tryptase (µg/l)	Allergy/Autoimmunity	Enrolled in the clinical trial
**3**	M	27	0.1	YES	3.0	77.0	181	ND		No
**7**	M	44	20.0	YES	338	78	171	7.07		No
**12**	F	35	2.0	YES	9.8	60.9	155	10.30	House Dust mites, molds	No
**13**	F	63	0.6	YES	16.6	66.0	160	ND		No
**19**	F	49	0.8	YES	4.89	62.7	159	11.80	Anti-TPO, ANA positive	No
**22**	F	50	1.0	NO	58.3	63.3	163	3,8		No
**23**	M	44	15.0	YES	23.0	NR	NR	6.63	House Dust mites	No
**24**	F	55	3.0	YES+ DPU	2.6	92.0	165	5.80		No
**25**	F	64	0.1	YES	114	NR	154	7.73		No
**26**	F	58	0.6	YES	101.0		163	5.38	Sensitized to anisakis	No
**27**	F	40	1,5	YES	21.5	69.7	171	4.23	Pollen/Anti-TPO positive	No
**28**	M	27	4.0	YES	173.0	91.0	186	6.44		No
**29**	M	58	0.9	YES	208.0	NR	NR	4.79	Sensitized to anisakis	No
**30**	M	53	3.0	NO	48.0	74.0	177	4.73	Pollen	No
**33**	F	33	1.5	YES	19.2	69.0	176	ND	Pollen/Anti-TPO positive	No
**38**	F	37	0.6	NO	7.0	NR	NR			No
**47**	M	32	17.0	YES	166	NR	NR	10.20	House Dust mites, grass	No
**48**	F	17	8.0	YES	2563.0	66	161	2.97	Pollen, house dust mite, nuts	No
**49**	F	48	4.0	NO	57	NR	NR	5.7		No
**52**	F	45	2.0	YES	281.0	NR	NR	<11		No
**53**	M	36	7.0	YES	14.6	NR	NR	ND		No
**54**	F	37	6.0	YES	11.6	NR	NR	ND		No
**101**	M	47	1.0	YES	73.3	94.0	175	3.50	Rhinitis	Yes
**102**	M	26	7.0	YES	26.0	106.5	195	1.38	Rhinitis	Yes
**103**	F	69	1.0	NO	76.7	60.4	146	2.96		Yes
**105**	F	49	40.0	YES	2.0	69.4	158	4.86	Hyperthyroidism (TSH; T4)	Yes
**106**	F	39	3.0	YES	73.0	63.5	156	1.58		Yes
**108**	M	46	2.0	YES	28.3	96.9	181	2.37		Yes
**109**	F	31	14.0	YES	19.8	61.2	167	1.23		Yes
**110**	M	55	1.0	YES	25.4	93.0	173	3.20	Rhinitis	Yes
**111**	M	44	10.0	YES	471.0	106.0	177	1.88		Yes
**112**	M	44	2.0	YES	429.0	86.0	171	1.53	Rhinitis	Yes
**113**	F	31	2.0	NO	55.1	50.0	160	2.67	Rhinitis/Asthma/Autoimmune Hyperthyroidism/ Celiac	Yes
**114**	F	46	24.0	NO	534.0	61.6	157	2.34	Autoimmune Hyperthyroidism	Yes
**115**	M	57	2.0	NO	NR	NR	NR	1.98	Vitiligo	Yes
**117**	M	52	24.0	YES	125.5	66	160	1.85	Rhinitis	Yes
**118**	M	43	15.0	YES	3.8	92	179	2.63	Diabetes/Anti-TPO positive	Yes
**119**	F	72	1.0	YES	7.2	83	166	6.20	Hyperthyroidism (TSH, T4)	Yes
**120**	F	36	1.0	YES	22.1	120	167	<1.00	Rhinitis	Yes

ND: Not Determined, NR: Not registered, DPU: Delayed pressure urticaria

Supplementary Table 2A. Differences in the CU-Q2oL score, UAS and VAS score before and after omalizumab treatment

	Baseline	Omalizumab	Within-individual difference	p-value
Overall score	22.8 (8.7, 34.8)	6.5 (3.3, 15.2)	-5.4 (-29.3, 1.1)	0.047
Pruritus	50 (25, 62.5)	25 (0, 25)	-25 (-50, 0)	0.008
Swelling	12.5 (0, 25)	0 (0, 25)	0 (-25, 0)	0.332
Impact on life activities	20.8 (4.2, 41.7)	0 (0, 8.3)	-4.2 (-37.5, 0)	0.012
Sleep disorders	25 (15, 35)	10 (0, 25)	-5 (-30, 5)	0.123
Limitations	16.7 (8.3, 25)	0 (0, 25)	0 (-25, 0)	0.263
Appearance	20 (0, 30)	0 (0, 10)	0 (-20, 0)	0.156
Urticaria activity score (UAS)	3 (2, 6)	0 (0, 2)	-2 (-4, 0)	0.004
Itching	2 (1, 3)	0 (0, 1)	-1 (-3, 0)	0.001
Wheals or hives	1 (1, 3)	0 (0, 1)	-1 (-2, 0)	0.019
Visual analogue scale (VAS) score	60 (25, 75)	10 (1, 29)	-23 (-53, -7)	0.001

Supplementary Table 2 B. Differences in the CU-Q2oL score, UAS and VAS score before and after placebo treatment

	Baseline	Placebo	Within-individual difference	p-value
Overall score	22.8 (7.6, 34.8)	12.0 (2.2, 27.7)	-4.9 (-16.8, 3.3)	0.079
Pruritus	37.5 (12.5, 50)	37.5 (25, 75)	-12.5 (-25, 0)	0.133
Swelling	0 (0, 12.5)	6.3 (0, 25)	0 (0, 12.5)	0.227
Impact on life activities	25 (0, 41.7)	4.2 (0, 16.7)	-8.3 (-25, 0)	0.015
Sleep disorders	25 (5, 35)	10 (0, 25)	-5 (-20, 0)	0.147
Limitations	16.7 (0, 25)	8.3 (0, 41.7)	0 (-8.3, 8.3)	0.884
Appearance	15 (5, 25)	10 (0, 25)	-5 (-10, 0)	0.181
UAS	3 (1, 5)	2 (0, 3)	-2 (-3, 1)	0.082
Itching	2 (1, 3)	1 (0, 2)	-1 (-2, 0)	0.074
Wheals or hives	1 (1, 2)	1 (0, 1)	-1 (-2, 0)	0.110
VAS score	48 (9, 72)	27 (4, 60)	-11 (-45, 21)	0.344

*Values expressed as medians (25th percentile, 75th percentile).

**Scores were transformed to a 0 to 100 scale. Higher scores represent worse health-related quality of life.

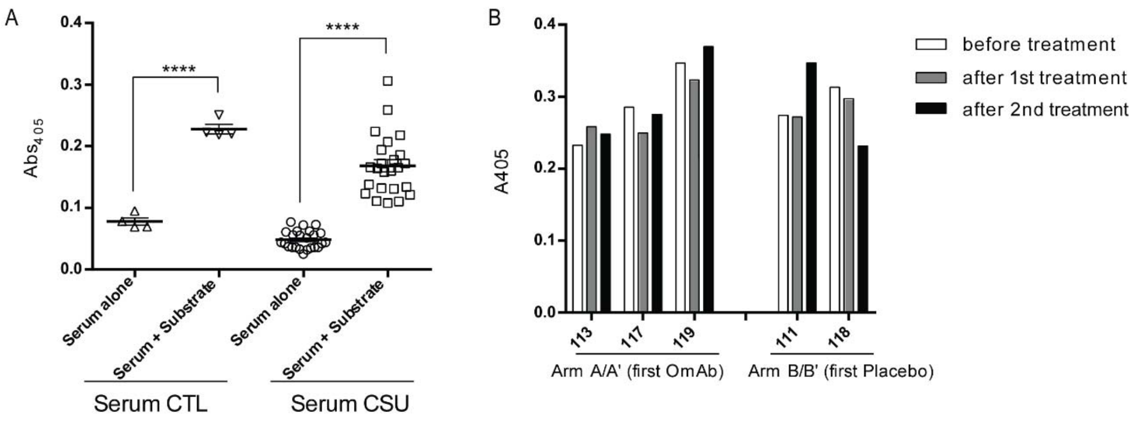

Figure S1: Sera from CSU patients show intrinsic β-hexosaminidase activity that does not change after OmAb/Placebo treatment.

A) Intrinsic β-hexosaminidase activity in sera from healthy controls (CTL) and chronic spontaneous urticaria patients (CSU) was evaluated by incubation of sera with β-hexosaminidase substrate and compared with sera in absence of enzymatic substrate. Statistical significance (****$p < 0.0001$) was determined (unpaired two-tailed T-test (CI:95%)). B) The charts represent the intrinsic β-hexosaminidase activity in sera from CSU patients at different time points of the OmAb/Placebo treatment.
